# Carbon Based Polymeric Nanocomposite Hydrogel Bioink: A Review

**DOI:** 10.3390/polym16233318

**Published:** 2024-11-27

**Authors:** Alle Madhusudhan, Tejaskumar A. Suhagia, Chhavi Sharma, Saravana Kumar Jaganathan, Shiv Dutt Purohit

**Affiliations:** 1 Department of Chemistry, The University of Memphis, Memphis, TN 38152, USA; tejaskumarsuhagia@gmail.com; 2 Department of Biotechnology, University Centre for Research and Development, Chandigarh University, Mohali 140413, Punjab, India; chhavi.e15032@cumail.in; 3 Institute of Research and Development, Duy Tan University, Da Nang 550000, Vietnam; 4 School of Engineering & Technology, Duy Tan University, Da Nang 550000, Vietnam; 5 School of Engineering, College of Health and Science, Brayford Pool, Lincoln LN67TS, UK; 6 Department of Biomedical Engineering & Biotechnology, Khalifa University of Science and Technology, Abu Dhabi 127788, United Arab Emirates

**Keywords:** nanocomposite hydrogels, graphene, carbon nanotubes (CNTs), carbon dots (CDs), activated charcoal (AC)

## Abstract

Carbon-based polymeric nanocomposite hydrogels (NCHs) represent a groundbreaking advancement in biomedical materials by integrating nanoparticles such as graphene, carbon nanotubes (CNTs), carbon dots (CDs), and activated charcoal (AC) into polymeric matrices. These nanocomposites significantly enhance the mechanical strength, electrical conductivity, and bioactivity of hydrogels, making them highly effective for drug delivery, tissue engineering (TE), bioinks for 3D Bioprinting, and wound healing applications. Graphene improves the mechanical and electrical properties of hydrogels, facilitating advanced tissue scaffolding and drug delivery systems. CNTs, with their exceptional mechanical strength and conductivity, enhance rheological properties, facilitating their use as bioinks in supporting complex 3D bioprinting tasks for neural, bone, and cardiac tissues by mimicking the natural structure of tissues. CDs offer fluorescence capabilities for theranostic applications, integrating imaging and therapeutic functions. AC enhances mechanical strength, biocompatibility, and antibacterial effectiveness, making it suitable for wound healing and electroactive scaffolds. Despite these promising features, challenges remain, such as optimizing nanoparticle concentrations, ensuring biocompatibility, achieving uniform dispersion, scaling up production, and integrating multiple functionalities. Addressing these challenges through continued research and development is crucial for advancing the clinical and industrial applications of these innovative hydrogels.

## 1. Introduction

In recent years, the field of biomedical materials has witnessed significant advancements, particularly due to innovations in nanotechnology. Among the most promising developments is the emergence of carbon-based polymeric nanocomposite hydrogels (NCHs) [1]. These hydrogels, which incorporate nanoparticles such as graphene, carbon nanotubes (CNTs), carbon dots (CDs), and activated charcoal (AC) into traditional hydrogel matrices, have demonstrated enhanced mechanical, electrical, and biological properties, making them ideal candidates for advanced biomedical applications [2]. Importantly, their unique properties have garnered increasing attention in 3D Bioprinting, where precision, biocompatibility, and mechanical integrity are essential for replicating complex tissue architectures.

Graphene-based hydrogels have emerged as a particularly transformative material in 3D Bioprinting due to their exceptional mechanical strength, electrical conductivity, and surface area. The integration of graphene into hydrogels enables the fabrication of scaffolds that more closely mimic the structural and functional properties of native tissues [3]. These properties are crucial for applications in tissue engineering (TE), particularly in the creation of biomimetic scaffolds that require electrical stimulation, such as neural and cardiac tissues [4]. In 3D Bioprinting, graphene-enhanced hydrogels offer superior printability, structural integrity, and functionality, making them highly effective in tissue scaffold engineering [5].

Similarly, carbon nanotubes (CNTs) bring added value to 3D bioprinting applications. CNTs, whether single-walled (SWCNTs) or multi-walled (MWCNTs), are renowned for their high mechanical strength, electrical conductivity, and flexibility. Their incorporation into hydrogel bioinks enhances the rheological properties, facilitating the 3D printing of intricate, high-fidelity tissue structures [6]. The conductivity of CNTs is especially beneficial in bioprinting tissues like neurons, bones, and muscles, where electrical signals play a critical role in cell function and tissue regeneration [7]. CNT-based hydrogels not only support structural complexity but also maintain bioactivity, which is crucial for cell growth and tissue integration.

Carbon dots (CDs) also play a role in advancing 3D Bioprinting, particularly in theranostic applications, where bioinks must simultaneously support tissue growth and enable real-time imaging. The fluorescent properties of CDs can be utilized in Bioprinting to track scaffold development and tissue integration, providing both functional and diagnostic benefits without compromising biocompatibility [8].

Activated charcoal (AC), with its high surface area and adsorption properties, further enhances the mechanical and antibacterial properties of NCHs, making them suitable for creating bioprinted scaffolds with improved bioactivity and infection resistance [9]. In 3D Bioprinting, AC-enhanced hydrogels offer the potential to create scaffolds that are not only mechanically robust but also electroactive, opening new avenues for bioprinted tissues requiring electrical stimulation or biosensing capabilities.

This review aims to explore the role of carbon-based nanocomposite hydrogels in advancing 3D bioprinting applications. By focusing on the integration of graphene, CNTs, CDs, and AC into hydrogel bioinks, the review highlights their contributions to improving the structural, electrical, and biological functionalities necessary for fabricating complex tissue scaffolds. Addressing the challenges of nanoparticle dispersion, biocompatibility, and scaling in 3D Bioprinting is essential to unlock the full potential of these innovative materials in TE and regenerative medicine.

## 2. Unique Characteristics of Carbon-Based Polymeric Nanocomposite Hydrogels (NCHs)

Due to their exceptionally high moisture content, hydrogels are incredibly soft and biocompatible. They have grown to be a significant soft material with a broad range of uses in industries like electrochemistry, biomedicine, and bionic smart materials. In order to improve stability and strength, NCHs are regarded as a group of materials that use nanoparticles as a reinforcing phase and polymers as a matrix [10]. NCHs are formed by combining hydrophilic polymer networks with physical crosslinks or covalent solid chemical connections with nanoparticles to create complex structures at the nanoscale [10]. NCHs networks can be constructed from a wide range of natural (organic) and synthesized hydrogels. Hydrogels with nanocomposite structures have improved rheological properties, as well as increased structural strength [11], tensile transparency strength, fracture energy, compressive strength, modulus elasticity, swelling and releasing rate, thermal conductivity, heat capacity, and torsional strength [10]. A range of organic or inorganic nanoparticles, referred to as nanofillers, are combined with the hydrogel matrix to create NCHs. This greatly improves the hydrogel matrix’s internal and external characteristics. Additionally, nanoparticles can function as crosslinkers that initiate in-situ free radical polymerization by interacting with initiator molecules and helping to fortify and harden the hydrogel [10].

The creation of nanocomposite hydrogels involves various nanomaterials such as polymeric nanoparticles, metal/metal oxide nanoparticles, carbon-based nanomaterials, and inorganic nanoparticles [12]. Carbon-based nanomaterials, in particular, are used to enhance the mechanical, optical, and electrical properties of hydrogels [13]. Carbon nanotubes (CNTs) are a prominent example of such nanomaterials. Their use as nanofillers in hydrogels is attributed to their superior electrical conductivity, stable electrochemical properties, low density, high mechanical strength, and extensive surface area [14]. Recent studies highlight the adaptability of CMs in hydrogels, where they are used to adjust rheological characteristics, enhance mechanical properties, and introduce new physical features such as roughness, shape memory, and conductivity. These modifications influence the biological behaviour of bioprinted products [14]. The incorporation of CMs into 2D hydrogels has been shown to improve attributes such as electrical conductivity, fluorescence, mechanical strength, shape memory, biosensing capabilities, and self-healing properties [15]. The resulting composite hydrogels exhibit unique properties based on the type and characteristics of the CMs used, making them suitable for various biological applications [2,16]. This section provides an overview of the primary characteristics of carbon-based materials, focusing on their attributes and recent advances in 3D Bioprinting with CMs.

To date, research has primarily explored hydrogels combined with CNTs, graphene, carbon nanodots, and activated charcoal for 3D bioprinting applications.

Graphene oxide (GO) and carbon nanotubes (CNTs) are the primary carbon materials employed in hydrogel formulations. The most investigated CM for creating hydrogel composites for Bioprinting has been graphene and, specifically, GO [2]. The combination of carbon materials with hydrogels is gaining interest due to the CMs’ inherent properties and versatility. This trend is driven by the need for biomimetic structures that closely mimic native tissues, which has led to the development of specialized hydrogel constructs capable of controlling cell behavior through 3D bioprinting techniques [17]. The material’s remarkable properties, such as its nanoscale thickness and micrometer lateral size, made it the thinnest 2D filler to date. Graphene’s structure allows it to have exceptional mechanical, electrical, and thermal properties, but when combined with other materials, its interfacial bonding with polymer matrices remains difficult due to its surface chemical [2]. To get around this problem, chemical oxidation has been used to create graphene oxide (GO) [2], which induces the formation of reactive oxygen functional groups while sacrificing electrical conductivity.

Because of its chemical adaptability, GO has been employed in a variety of applications, such as carbonyl groups (CO), epoxy (-COC), hydroxyl (-OH), and carboxyl (-COOH). Its combination with various materials, such as hydrogels, has consequently made it possible to create nanocomposites with improved qualities, like improved mechanical characteristics and thermoresponsive behavior [18]. Due to the high concentration of COC, OH, COOH, and CO groups in GO, the interfacial interactions between these materials can be of many different types, such as covalent bonding, hydrogen bonding, electrostatic interaction, and sterification (polymers with a carboxyl terminated group that can crosslink with GO hydroxyl groups) [2].

Carbon dots (CDs) are multipurpose nanomaterials that are widely employed in biomedicine, environmental applications, food, and agriculture. They have antimicrobial qualities, fluorescence stability, and adsorption capability. CD-based hydrogels combine the advantages of both materials by enhancing hydrogels’ mechanical, self-healing, and adsorption qualities and adding novel antibacterial and fluorescent qualities. Additionally, the increased fluorescence stability of CD-based hydrogels guards against the quenching of fluorescence brought on by CD aggregation [19]. Additionally, they have low production costs, weak protein interactions, easy body clearance, good biocompatibility and permeability, and other characteristics that make carbon nanodots excellent platforms for drug delivery or imaging agents in multifunctional theranostic applications like gene therapy and chemotherapy.

Activated charcoal (AC), an amorphous CM that has undergone chemical modification to create a microporous three-dimensional structure, is another kind of CM. The surface functionality of AC, which is defined by a high adsorption capacity of numerous chemical species, is one of its primary characteristics. It is a helpful adsorbent in industrial operations as well as biomedical applications (cholesterol level management, poisoning, etc.) due to its high surface area-to-volume ratio [20]. AC has also been described as a catalyst that promotes the production of radicals, which is crucial for oxidizing pigments that come from industrial processes.

Since CMs are renowned for their exceptional qualities, there has been much research into using them to create novel hydrogel-based composites that provide electrical conductivity and mechanical support. Table 1 summarizes some of the key features of carbon-based materials.

### 2.1. Mechanical and Rheological Properties

The mechanical performance of hydrogels is enhanced by the inclusion of CMs. Since hydrogels usually have minimal mechanical qualities, this is a huge advantage. Furthermore, CMs have the ability to interact with the functional groups of polymers, accelerating crosslinking and minimizing the need for extra crosslinking procedures [28].

Carbon-based nanocomposite materials account for approximately 20% of all nanocomposite hydrogels. Their superior mechanical strength, optical qualities, and electrical conductivity make carbon-based nanoparticles popular choices in hydrogel architectures [10]. The enhanced mechanical strength and toughness result from the force and stress distribution facilitated by covalent crosslinks between nanocarbon compounds and polymer matrices [10]. The rheological characteristics of the inks are altered when CMs are present. The type and quantity of CMs produce varying rheological characteristics. This implies that the ink viscosity of the hydrogels can be adjusted to produce the ideal printing conditions both during and after printing [29].

Graphene, composed of a single layer of carbon atoms arranged in a two-dimensional honeycomb lattice, possesses remarkable properties: a specific surface area of 2630 m^2^/g and a Young’s modulus of approximately 1.0 ± 0.1 TPa. However, graphene’s hydrophobic nature limits its use in water-based systems and hydrophilic compounds such as hydrogels [26,30,31,32].

Graphene oxide (GO), which contains oxygen-based functional groups like hydroxyl (-OH), alkoxy (C-O-C), and carbonyl (C=O), addresses these limitations [32]. GO’s single-layer morphology and functional groups, such as epoxy and carboxyl groups on the basal plane and hydroxyl and carbonyl groups at the edges, enhance its dispersion in aqueous solutions [26]. GO’s thermal and electrical conductivity differs from graphene due to the presence of these functional groups [32]. A hydrogel developed from Pluronic F127-DMA as matrix and GO (0.2 wt%) MWCNTs (2 wt%) as filler materials by extrusion-based Bioprinting applying radical polymerization crosslinking approach has shown to possess improved hydrogel mechanical properties [29]. Such hydrogels are useful for applications from robots to TE. GO was utilized to increase the hydrogels’ mechanical strength (compressive elastic modulus reaching 112 kPa) and structural integrity in a different study by Valentin et al. [29] that showed the ionic crosslinking of alginate hydrogels. In another study, Alginate and GO (0.005–0.025% (*w*/*v*)) with CaCl_2_ as a crosslinker is known to improve ink rheological properties [33,34].

Carbon nanotubes (CNTs), with their unique cylindrical structure, offer flexibility, regular pore structures, wide aspect ratios, and low density, making them effective reinforcement materials for polymer nanocomposites [35]. CNTs are characterized by their hollow architecture, high electrical conductivity, tensile strengths between 50 and 150 GPa, and high Young’s modulus [36]. The two main types of CNTs are single-walled (SWNTs) and multi-walled (MWNTs), each with distinct properties influenced by their atomic structure [36].

CNTs may be used in Bioprinting for a variety of reasons. Enhancing the rheological and mechanical characteristics of biomaterial inks is one objective [2]. Enabling hydrogels electrical conductivity is another important application for CNTs [2].

For example, Liu et al. [37] demonstrated that incorporating 0.8 weight percent functionalized CWNTs in PVA nanocomposites increased tensile yield strength and modulus by 47% and 79%, respectively. Similarly, Huang et al. found that 1.0 weight percent MWCNTs significantly improved toughness and tensile strength in PVA hydrogels, showing increases of 63% and 13.333%, respectively [38]. In another study, the presence of CNTs, double network hydrogel poly (ethylene glycol)/Poly vinyl alcohol (PEG/PVA) with shape memory and appropriate printability was produced, demonstrating their ability to preserve the hydrogel’s shape memory performance and enhance its viscoelasticity, which improves with increasing CNT concentrations [39]. The MWCNTs were covalently bonded to poly (ethylene glycol) diacrylate [PEGDA] using amines to guarantee even dispersion throughout the polymer matrix, as 0.1% MWCNTs were used. The mechanical characteristics improved by 189% as compared to controls (E = 1.1 ± 0.7 MPa) [40]. In another study, Khabibullin et al. created hydrogels for TE with chemical and biological sensing capabilities by combining carbon quantum dots with cellulose nanocrystals. Carbon nanodots (QDs) were employed as crosslinkers to modify the hydrogels’ rheological characteristics. This was made possible by the hydrophobic contacts and hydrogen bonds that predominate and overcome the electrostatic repulsion between these nanoparticles [41].

Additionally, it was shown that the hydrogel maintained its structure after extrusion, adhering to the predetermined pattern at a QD dosage of 10 mg/mL. The hydrogels were luminous when exposed to light excitation at 365 nm [41]. When the created hydrogels offer advantages over conventional medicines, they may concurrently deliver targeted therapy and fluorescence-based diagnosis, opening up new insights in the theragnostic sector.

Carbon nanodots (QDs) have also been used to adjust hydrogel rheological properties through hydrophobic interactions and hydrogen bonding, overcoming electrostatic repulsion between nanoparticles. These QDs can maintain hydrogel structure upon extrusion and exhibit fluorescence under light excitation at 365 nm, offering the potential for targeted therapy and diagnostics [41].

### 2.2. Adjustable Conductivity, Swelling, Optical, and Thermoresponsive Properties

The addition of GO significantly impacts the swelling ratios, kinetics, and responsiveness of polymeric matrices. Swelling ratios typically increase with GO fractions up to 0.3 weight percent before declining [42]. The reduction of GO to partially recover the graphene structure and SP^2^ hybridized carbons is achieved through techniques such as ultraviolet irradiation, high-temperature thermal annealing, and electrochemical methods [14,43]. These reduction methods influence the final material properties, making careful selection essential.

Reduced graphene oxide (rGO) exhibits improved optical and electrical conductivity and is better suited for incorporation into hydrogel nanocomposites. However, rGO’s hydrophobic nature and limited solubility in aqueous solutions can lead to poor stability and reduced surface area for molecular interactions, presenting challenges for various applications [43].

GO’s chemical versatility, with functional groups such as carbonyl (CO), carboxyl (–COOH), hydroxyl (–OH), and epoxy (–COC), allows for the creation of nanocomposites with enhanced mechanical and thermoresponsive properties [44]. These interactions can include covalent bonding, esterification, hydrogen bonding, and electrostatic interactions, each contributing to varying material qualities [45].

It has been found that PEGDA and amine-functionalized MWCNTs (0.02–0.1 weight percent) hydrogels can influence electrical conductivity, enhance mechanical qualities, and have applications in nerve TE and the applications employing electrical field [40].

MWCNTs were added to cellulose-based scaffolds with the goal of constructing electrically conductive scaffolds for neural applications. The electrical conductivity of 3.8 × 10^−1^ S/cm was attained upon the addition of 20 weight % CNTs. Furthermore, conductive CNTs encourage the spontaneous electrical activity of neurons even in the absence of external electrical stimulation, leading to improved neural network growth [46]. In another study involving the transformation of cellulose nanofibril hydrogel into three-dimensional structures with regulated topologies, Håkansson et al. found that the inclusion of CNTs enhanced the conductivity by three orders of magnitude, from 10^−6^ S/cm to 10^−2^ S/cm, even at low mass fractions (<6 wt%) [47]. The creation of patterns on flexible substrates like paper, elastomers, or hydrogels has also taken advantage of these characteristics in electrically conductive CNT-based inks. The produced GelMA/DNA-coated MWCNTs have a conductivity of 24 ± 1.8 S/cm and a viscosity of 0.01 Pa/s^−1^ at a shear rate of 50 s^−1^ that is appropriate for extrusion-based bioprinting [48].

Offering hydrogels electrical conductivity is another important application for CNTs. With the goal of fabricating an injectable biomaterial ink with shear thinning behavior, Deng et al. [49] created a thermoresponsive self-healing hydrogel composed of N-isopropyl acrylamide (NIPAM), nanoclay (laponite), and carbon nanotubes. The thermos-responsiveness was caused by the application of NIPAM, and the self-healing and adhesive qualities were also influenced by electrostatic interactions in the crosslinking agent lapotnite. With no effect on the stretchability of hydrogels, the addition of CNTs gave the scaffolds improved mechanical qualities (E = 46.8 kPa) and electrical conductivity, which might be used in sensors.

Wu et al. developed poly(N-acryloylglycinamide-poly(2-acrylamido-2-methyl-1-propanesulfonic acid)/poly(3,4-ethylenedioxythiophene)/polystyrene sulfonate (PNAGA-AMPS/PEDOT/PSS) hydrogels with AC in 3D arbitrary shapes [50]. The presence of AC gives the hydrogel a supercapacitor activity that results in printable electrodes and does not impair the biomaterial ink’s printability [50].

### 2.3. Biocompatibility

When CMs are present, hydrogels acquire new properties like roughness, shape memory, and electrical and thermal conductivity (if not oxidized). Although the processes behind these cell-instructive qualities are still unclear, it has been demonstrated that these characteristics alter the biological response of seeded cells. Applications in the heart, neurological system, bone, and cartilage were determined to be the primary focus of current research. Additionally, it has been shown that CMs can load medicines into hydrogel inks, making 3D structures appropriate for drug release systems. Thus, carbon-based materials (CMs) are extensively studied for their role as fillers or matrices in various polymeric composites, including hydrogels [2]. Their antibacterial properties, electrical and thermal conductivity, and adaptability for biofunctionalization make them ideal for developing sophisticated biomaterials with precise control over cell-material interactions [2].

Graphene oxide has been demonstrated to be compatible with the human body and is also a widely recognized drug nanocarrier. However, numerous variables and experimental setups affect how graphene nanoparticles influence cell survival [51]. As a result, in vitro cell cultures and in vivo animal models must be used to examine the toxicity and biocompatibility of graphene and its derivatives. Chang et al. discovered that GO exhibits no discernible cytotoxicity and does not penetrate A549 cells, which are human alveolar basal epithelial cells. However, at high concentrations, GO tends to cause a little decrease in cell viability by inducing dose-dependent oxidative stress in cells. Size and dosage have an impact on these outcomes [52], GO also shows dose-dependent toxicity to mice and human fibroblast cells, according to Wang et al. [53]. According to their findings, GO does not harm human fibroblast cells at concentrations below 20 μg/mL. Evident cytotoxicity, including decreased cell adhesion and cell death, is seen at concentrations greater than 50 μg/mL. Low doses (0.1 mg) and moderate doses (0.25 mg) of GO do not exhibit any overt toxicity in in vivo mouse experiments; nevertheless, a high dose (0.4 mg) results in chronic toxicity, which kills the animals and induces lung granuloma formation.

In vivo biodistribution of graphene functionalized with polyethylene glycol (PEG) in mice was studied by Yang et al. Under a dose of 20 mg/kg for three months, they showed that PEGylated graphene does not cause noticeable toxicity [54].

Carbon quantum dots (QDs), with diameters between 2 to 10 nm, exhibit intense fluorescence and are biocompatible, making them suitable for drug delivery and imaging applications [55,56]. Their low production cost, rapid clearance from the body, minimal protein interactions, and excellent biocompatibility enhance their potential for use in gene therapy and chemotherapy [55].

Carbon-based quantum dots also offer promising bioimaging, biosensing, and drug delivery features due to their low toxicity and excellent biocompatibility. Their exceptional electronic characteristics enable optronics, catalysis, and sensors applications through chemiluminescence and electrochemical luminescence [2].

Moreover, conductive bioinks incorporating nanomaterials have been developed to enhance signal transduction in embedded cells. For instance, CNTs dispersed in GelMA or HA with DNA as a surfactant resulted in biocompatible inks where cardiomyocytes showed 90% viability and normal morphology and elevated levels of cardiomyocyte-specific markers [57].

A study added MWCNTs to cellulose-based scaffolds to construct electrically conductive scaffolds for neural applications. This allowed for good filament width control (<1 mm) and increased cell growth [46]. Li et al. have reported encouraging outcomes with regard to the cardiac application [25]. The authors showed that CNTs, especially in concentrations less than 0.5 weight percent, greatly enhance the mechanical characteristics of 3D hollow structures while preserving the viability of fibroblasts, smooth muscle cells, and endothelial cells. Wu et al. fabricated PNAGA-AMPS/PEDOT/PSS hydrogels with AC in 3D arbitrary shapes [50]. The printability of biomaterial ink is unaffected by the presence of AC. Furthermore, these hydrogels can be used in a variety of electrical applications, such as electroactive scaffolds for soft TE, due to their biocompatibility (cell survival of 74–100% of L929 fibroblasts) [50].

## 3. Carbon-Laden Materials and Nanocomposite Hydrogel Ink for Bioprinting

Carbon-based materials are incredibly versatile, composed primarily of carbon atoms that can adopt various configurations, including 0D quantum dots, 1D carbon nanotubes (CNTs), 2D graphene, and 3D nanodiamonds. This versatility results in a broad spectrum of materials with distinct properties [40,58,59,60]. Carbon nanomaterials, in particular, have garnered significant attention across fields such as electronics, materials science, energy, and medicine due to their exceptional physical and chemical characteristics, including small size, high surface area, and superior mechanical, thermal, and electrical properties [40,58,59,60]. Ongoing research continues to develop new carbon nanomaterials to enhance the performance of both current applications and future technologies [61]. Additionally, carbon-based materials have found applications as bioinks for printing tissues and organs [59,62,63]. For example, graphene is widely used in bioink due to its outstanding electrical conductivity, mechanical strength, thermal conductivity, and support for cell growth. At the same time, carbon dots, known for their unique optical properties, are employed in drug delivery, bioimaging, sensing, and tissue regeneration [2,59,62,63].

Hydrogels are versatile materials utilized across various biomedical fields, including drug delivery, wound healing, TE, and antibacterial therapies. They exhibit key features such as biocompatibility, adjustable properties, and structural similarity to the natural extracellular matrix, making them invaluable in medicine. However, conventional hydrogels often lack the mechanical strength, electrical conductivity, and biological functionality needed for advanced TE applications [64]. This gap has prompted researchers to develop innovative hydrogel variations, such as nanocomposite hydrogels (NCHs), which enhance hydrogel properties by integrating nanoparticles into polymer networks.

These NCHs can be categorized based on the types of hydrogels they incorporate, including natural hydrogels (e.g., chitosan, alginate) and synthetic hydrogels (e.g., polyacrylamide, polyethylene glycol). The choice of hydrogel type influences their biocompatibility, mechanical properties, and potential applications. NCHs are formed by embedding nanoparticles into hydrophilic polymer networks through either covalent bonds or physical crosslinks, resulting in complex nanoscale structures. The incorporation of nanoparticles—whether carbon-based, polymeric, or inorganic—can improve various hydrogel characteristics such as structural integrity, transparency, compressive strength, elasticity, substance absorption/release, and thermal properties [65].

These features make NCHs adaptable for specific biomedical applications. Hydrogels consist of flexible polymeric networks that form intricate three-dimensional (3D) structures with nanoscale interstitial spaces, serving as hosts for various nanomaterials, known as guests [66]. When incorporating nanomaterials like carbon-based nanoparticles (e.g., carbon nanotubes, graphene) or metal nanoparticles into these polymeric networks, these nanomaterials act as “guests”, resulting in nanocomposite hydrogels. The interactions between the hydrogel matrix and these nanoparticles include van der Waals forces, hydrogen bonding, electrostatic interactions, π-π stacking, and covalent bonding [66]. Functionalized nanoparticles enhance the mechanical and biological properties of hydrogels, while non-functionalized carbon-based nanoparticles, such as graphene and carbon nanotubes, generally rely on π-π stacking for interaction [66]. This interaction enhances the hydrogels’ properties compared to their non-nanocomposite counterparts.

Furthermore, 3D Bioprinting has transformed TE by enabling the creation of complex structures that mimic biological tissues. This technique allows for the precise deposition of biomaterials, cells, and bioactive molecules to fabricate tissue-like structures, and integrating carbon materials into hydrogels has become a focus of bioprinting research, enhancing properties such as mechanical strength, electrical conductivity, fluorescence, and self-healing capabilities [2]. Key carbon materials used include graphene and its derivatives (e.g., graphene oxide, reduced graphene oxide), carbon nanotubes, nanodiamonds, fullerenes, and carbon-based quantum dots. Various 3D printing methods—such as extrusion-based direct ink writing, volumetric Bioprinting, particle fusion-based printing, light-induced methods, and inkjet writing—are employed to create complex 3D structures. Direct ink writing is particularly popular due to its ease of use, cost-effectiveness, and scalability, as it involves directly extruding customized materials to create patient-specific objects [67]. However, the limited variety of available printing inks remains a challenge for widespread adoption, as these inks need to be biocompatible, biodegradable, and exhibit suitable rheological properties to ensure effective extrusion and post-printing shape retention.

The following subsection will discuss the 3D bioprinting applications of carbon quantum dots, carbon nanotubes/nanorods, and activated charcoal in their bioinks and as hydrogels.

### 3.1. Carbon Quantum Dots

Carbon nanomaterials in the size range of 2 to 10 nm are referred to as carbon nanodots or carbon quantum dots (QDs). These quasi-spherical 0D materials primarily consist of sp^3^ hybridized carbon atoms and often possess an amorphous structure. Carbon quantum dots can be synthesized using top-down or bottom-up approaches from organic materials or bioinorganic carbon precursors. One of their most intriguing properties is intense fluorescence emission, with spectra ranging from 330 to 475 nm. Table 2 provides a summary of these optical properties and the results from various studies, demonstrating their effectiveness in applications such as 3D printing, in vivo imaging, and bioimaging diagnostics. Additionally, these materials are cost-effective to produce, exhibit low interactions with proteins, are easily removed from the body, and possess high biocompatibility and permeability. These characteristics make carbon nanodots excellent candidates for multifunctional theranostic applications, such as drug delivery and imaging agents, including their use in bioink for 3D bioprinters.

For instance, photoinduced atom transfer radical polymerization, a method for creating polymer networks, often suffers from low polymerization rates, limiting its full utilization in 3D printing [68]. However, the introduction of carbon quantum dots (CQDs) has enabled high monomer conversion rates (>90%) during polymerization, resulting in good dimensional accuracy of 3D-printed objects within 1 min under visible light. Additionally, CQDs impart photoluminescence to the printed objects, aiding in disease diagnosis [68].

**Figure 1:**
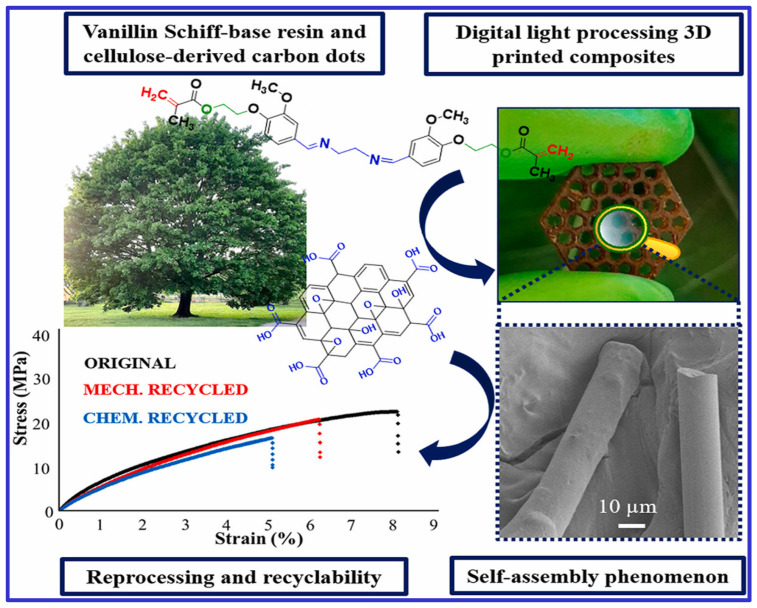
A recyclable composite formation with self-assembly of carbon dots during digital—processing 3D printing of vanillin Schiff-base resin.

Liguori et al. [69] investigated the self-assembly of carbon dots in a vanillin Schiff-base resin-based 3D printing ink. These carbon dot-based composites were formed into fibers embedded in a thermoset matrix using 3D printing with digital light processing, demonstrating both physical and chemical recyclability, as shown in Figure 1. Incorporating carbon dots enhanced the retention of the material’s characteristics throughout the recycling process.

Recently, Lee and colleagues [63] used carbon dots to develop a bone substitute for osteoclast inhibition and fluorescence imaging in 3D printing. Their fabricated bone substitute decreased osteoclast viability following transplantation, allowed for fluorescent in vivo imaging, and facilitated medication release through pH manipulation. The lack of selectivity and specificity in fluorescent materials for bone imaging remains a significant challenge in biomedical research. These findings suggest that bone loss treatments could become more precise, less invasive, and associated with fewer adverse effects. Similarly, Mahmud and coworkers [62] utilized carbon quantum dots in PLA to create 3D-printed nanocomposites to resist in-stent restenosis and stent thrombosis in cardiovascular systems. Their research showed that incorporating carbon quantum dots in PLA improved hydrophilicity, processability, mechanical strength, radial stability, and cell proliferation, significantly enhancing stent properties for cardiovascular applications with non-invasive imaging.

Carbon dots (CDs) are a newly discovered type of carbon-based nanomaterial, typically smaller than 10 nm, and are closely related to nanoparticles. CDs have garnered significant attention in TE and advanced biomedical applications due to their adjustable photoluminescence, excellent biocompatibility, enhanced mechanical properties, high solubility in water, and antibacterial characteristics. They are easily surface-modified, cost-effective, and exhibit low toxicity, making them ideal for imaging applications [55]. CDs have minimal protein affinity and can be synthesized from organic materials like CNTs, graphene, aromatic compounds, or bioorganic precursors such as food waste and silk.

CDs have primarily been studied in the context of extrusion-based Bioprinting. Khabibullin et al. developed hydrogels using cellulose nanocrystals and carbon quantum dots tailored for chemical and biological sensing in TE applications [41]. The carbon quantum dots acted as crosslinkers, adjusting the rheological properties of the hydrogels through hydrogen bonding and hydrophobic interactions. Experimental results showed that hydrogels containing 10 mg/mL of quantum dots retained their structural integrity and pattern adherence during extrusion. These hydrogels also exhibited fluorescence when excited at 365 nm, demonstrating their potential for theranostic applications, where diagnostic fluorescence imaging is combined with targeted therapeutic treatments [41]. This represents a notable advancement, highlighting the innovative use of CDs in Bioprinting.

### 3.2. Graphene-Based Nanomaterials

Graphene is a two-dimensional material characterized by a single layer of carbon atoms arranged in a hexagonal honeycomb lattice. It can also be produced in multilayered sheets with up to ten or more layers. Graphene is distinguished by its exceptional properties, including high thermal conductivity, flexibility, superior mechanical strength, low weight, and outstanding electrical conductivity. Remarkably, graphene is approximately five times lighter than aluminum and 200 times stronger than steel [61,70].

There are various types of graphene, each with unique properties: monolayer graphene exhibits exceptional electronic characteristics; bilayer graphene consists of two stacked layers; graphene oxide (GO) contains oxygen functional groups; reduced graphene oxide (rGO) is a reduced form of GO; graphene nanoribbons demonstrate unique electronic and magnetic behaviors; graphene quantum dots (GQDs) show quantum confinement effects; and 3D graphene, which is formed by stacking graphene sheets, offers enhanced mechanical strength and electrical conductivity. Due to these exceptional properties, graphene and its derivatives are of great interest in energy, electronics, sensors, composites, construction, and biomedicine fields.

This section focuses on the recent applications of graphene-based nanomaterials in the development of nanocomposite bioinks for 3D Bioprinting, particularly in clinical settings. Bioprinting, with its ability to assemble living cells, biomolecules, and biomaterials into three-dimensional structures, has emerged as a powerful platform for TE. Hydrogels, a commonly used biomaterial, are increasingly being developed into bioinks for the 3D printing of various human tissues and organs [61,70].

Among the various hydrogels, gelatin and GelMA (gelatin methacrylate) have garnered significant attention for 3D Bioprinting due to their biodegradability, intrinsic bioactivity, excellent biocompatibility, and the ability to undergo thermoresponsive gelation and chemical crosslinking. However, the low electroactive properties of gelatin and GelMA-based hydrogels limit their use in neuronal TE, where electrical conductivity is crucial. To address this issue, researchers have incorporated small amounts of highly electroactive GO into these hydrogels to create electroactive hydrogels suitable for 3D bioprinting neural tissues. GO nanoparticles impart shear-thinning behavior, adequate viscosity, and improved mechanical and electrical conductivity to the hydrogels, enabling the 3D printing of porous scaffolds with good structural integrity. Furthermore, GO-based bioinks, combined with mesenchymal stem cells derived from rat bone marrow, have been successfully 3D printed, demonstrating high cell viability and protection against UV exposure, attributed to the UV-shielding effect of GO nanoparticles [71].

In another study, Khabibullin et al. [41] developed a chemically and biologically responsive hydrogel by incorporating cellulose nanocrystals and carbon quantum dots (CQDs), with CQDs acting as crosslinkers to adjust the hydrogel’s rheological properties. The hydrogels maintained their structural integrity post-extrusion, following the predesigned pattern, with a CQD concentration of 10 mg/mL. Notably, these hydrogels exhibited fluorescence under 365 nm light, offering potential for theranostic applications by combining targeted therapy and diagnostics through fluorescence.

Further advancements include the development of GO composites loaded with human mesenchymal stem cells, which were cultured in 3D bioprinted bioreactors capable of withstanding mechanical loads for up to 56 days [72]. The study showed that continuous mechanical stress led to significant increases in organoid mineral density, rigidity, osteoblast differentiation, and the formation of a lacunar-canalicular network by day 56, highlighting the potential of these 3D bone organoids as human-specific models for drug screening and bone pathology research. Jiang and colleagues [73] proposed a method to create scaffolds for bone regeneration by combining GO and mesenchymal stem cells (MSCs) with GelMA as a bioink. The study demonstrated that incorporating GO into the bioink did not adversely affect the viability or printability of GelMA. Instead, it enhanced MSC proliferation and osteogenic differentiation, as evidenced by increased expression of osteogenesis-related genes and proteins, such as RUNX2, osteopontin, and osteocalcin.

Another research group [74] explored the use of alginate hydrogel as a base for 3D bioprinting inks. While alginate has limited cell affinity due to a lack of functional groups, GO can support various cell types and has potential applications in repairing heart, brain, and bone tissues. By blending GO with alginate and gelatin, the researchers created a bioink that demonstrated enhanced cell affinity and viability, making it suitable for 3D printing cell-supportive scaffolds.

Dorishetty et al. [75] investigated the effects of rGO and isopropyl alcohol (IPA) on silk fibroin-based bioinks for 3D printing. Their findings revealed that dispersing rGO in the silk fibroin matrix using aqueous IPA increased the hydrogel’s pore size and water absorption capacity. However, the addition of IPA also reduced the printing accuracy by affecting viscosity and contact angle. Despite these challenges, the rGO-enhanced hydrogels exhibited improved crosslink density, beta-sheet content, and mechanical properties while maintaining biocompatibility, even with increased rGO concentrations.

**Figure 2:**
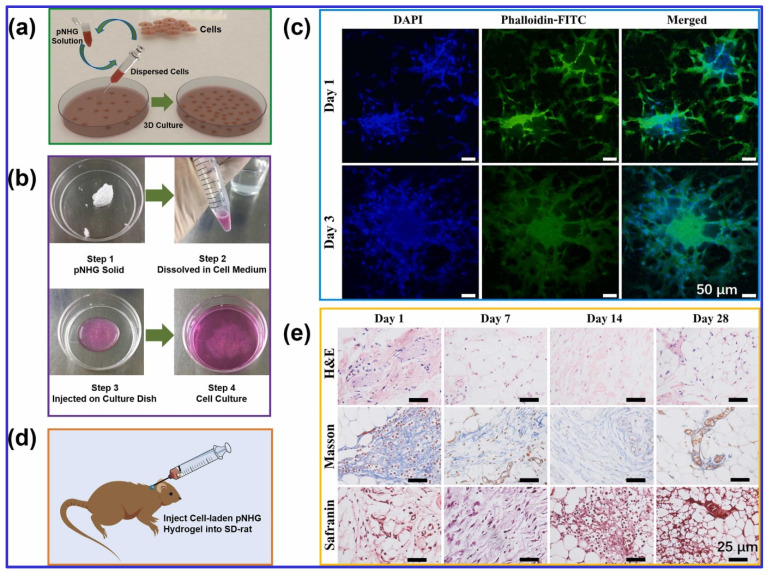
(a) The illustration of cell-laden pNHG hydrogel preparation. (b) The white pNHG solid powder is dissolved in cell solution (around 20 °C) first, and the cell-loaded solution is transferred to a Petri plate, then the Petri plate is put in a cell incubator with 95% air and 5% CO_2_ at 37 °C. Once the cell-laden hydrogel is formed, more cell medium is added for further cell growth. (c) Fluorescent images of hBMSCs encapsulated in pNHG2 hydrogels over days; the cells are stained with DAPI (blue) and Phalloidin-FITC (green). (d) The illustration of hBMSCs-laden pNHG2 hydrogel injected in the neck of SD rat. (e) Optical micrographs of H&E, Masson, and Safranin staining slices of surrounding tissues after injection of hBMSCs-laden pNHG2 hydrogel subcutaneously over days. Adapted with permission from [17]. Copyright Elsevier 2022.

Finally, temperature-responsive hydrogel bioinks show significant promise for cell encapsulation in TE and 3D Bioprinting. However, the stiffness of these hydrogels and the resulting cell aggregation often limit their applications. To overcome these challenges, Nie and coworkers [17] developed a novel temperature-responsive hydrogel composed of poly(N-isopropylacrylamide) and hydroxyethyl-chitosan, with dithiol-modified GO nanosheets. This hydrogel demonstrated excellent cytocompatibility with human bone marrow mesenchymal stem cells (hBMSCs) and allowed for cell loading at low temperatures (around 20 °C). The hydrogel provided a suitable environment for 3D cell culture, with good cell survival rates, suggesting its potential as a cell carrier in TE and drug delivery applications, as illustrated in Figure 2.

Graphene is a 2D material known for its remarkable mechanical, thermal, and electrical properties. Despite its potential, integrating graphene into hydrogels is challenging due to its surface chemical inertness. To overcome this, chemical oxidation is used to produce graphene oxide (GO), which has functional groups that can interact with hydrogels to form nanocomposite hydrogels with enhanced properties. Various crosslinking methods are used in bioprinting hydrogels with graphene, including radical polymerization and physical contact. Studies have shown that GO can improve hydrogels’ mechanical and thermal properties. For example, Cao et al. demonstrated that GO in PNIPAAm hydrogels could respond to near-infrared (NIR) stimulation, showing potential for 4D bioprinting applications like drug release systems [76]. Basu et al. used GO in extrusion-based Bioprinting to enhance the rheological properties and maintain hydrogel integrity during polymerization [29].

Other research has explored GO’s ability to create responsive hydrogels that change shape under various stimuli. For instance, Dai et al. synthesized a double network hydrogel with shape memory properties using GO and PLGA, useful for drug delivery systems [77]. Liu et al. and Li et al. studied the rheological behavior of hydrogels with GO, noting improvements in thixotropic properties and shape recovery [33,34]. Recent studies also highlight the use of GO in creating novel scaffolds for TE and drug delivery. For example, Cheng et al. developed a biomaterial ink for drug delivery targeting cartilage degeneration, and Zhou et al. used GO to enhance mechanical properties and chondrogenic differentiation in hydrogels. Despite its limitations in conductivity, graphene’s properties are explored for applications requiring electrical signaling, such as cardiac and neural TE scaffolds [78,79].

### 3.3. Carbon Nanotubes/Nanorods

Carbon nanotubes (CNTs) are cylindrical structures made up of one or more concentric layers of carbon atoms arranged in a hexagonal lattice. They are classified into two main types: single-walled nanotubes (SWCNTs), consisting of a single carbon layer rolled into a tube, and multi-walled nanotubes (MWCNTs), composed of multiple concentric cylinders separated by approximately 0.35 nm and interconnected by van der Waals forces [2,40,58,59,80]. CNTs are known for their exceptional mechanical, electrical, and thermal properties, comparable to graphene ones. Ideally, CNTs exhibit ballistic electron transport, minimizing heat dissipation [2]. They have emerged as a leading material for hydrogel composite bioprinting, with graphene as a notable alternative. Various bioprinting techniques, including extrusion-based and light-assisted methods, have been explored for incorporating CNTs into polymeric scaffolds [59,62,63,69].

Different polymer matrices have been utilized in hydrogel development, and no radical polymerization induced by light has been observed in extrusion-based Bioprinting. A primary goal of using CNTs in Bioprinting is to enhance the rheological properties of biomaterial inks. For instance, Nurly et al. investigated the incorporation of CNTs into a double network hydrogel (PEG/PVA), finding that CNTs improved the hydrogel’s shape memory and viscoelasticity, with performance-enhancing as CNT concentration increased [39].

Another significant application of CNTs is to impart electrical conductivity to hydrogels. Deng et al. [49] developed a shear-thinning biomaterial ink by combining N-isopropyl acrylamide (NIPAM), nanoclays, and CNTs. The NIPAM contributed thermoresponsive properties, while electrostatic interactions between laponite and the crosslinking agent influenced self-healing and adhesive properties. The addition of CNTs enhanced both the mechanical properties (E = 46.8 kPa) and electrical conductivity, making the scaffolds suitable for sensor applications.

Cui et al. [81] utilized MWCNTs in a polyion complex hydrogel for bone defect regeneration. Their hydrogel demonstrated good biocompatibility and supported osteogenic differentiation of rat bone marrow-derived mesenchymal stem cells (rBMSCs), leading to mineralized matrix formation and upregulation of osteogenesis-related genes. The MWCNT-laden hydrogel significantly facilitated the repair of calvarial defects in Sprague-Dawley rats.

Li et al. [80] explored MWCNTs in PEGDA hydrogels for chemical liquid detection. By incorporating 2 wt% MWCNTs, they reduced the electrical resistance of the hydrogel from 1.5 kΩ/m^−1^ to 0.022 kΩ/m^−1^. The hydrogel expanded upon exposure to chemical liquids, increasing its electrical resistance due to the loss of contact among MWCNTs during swelling. Similarly, Lee et al. [40] used amine-functionalized MWCNTs in a PEGDA matrix, achieving enhanced electrical properties (charge storage capacity increased from 0.133 ± 0.09 mC/cm^2^ to 2.21 ± 0.12 mC/cm^2^) and mechanical strength (increased by 189%) compared to controls. The MWCNT scaffolds also demonstrated high cell proliferation rates and supported neuronal and oligodendroglial differentiation in response to external electrical stimulation.

The potential of 3D Bioprinting for vascular applications has been explored due to its ability to reconstruct complex structures at a low cost [25]. Li et al. [25] developed a hybrid bioink combining CNTs, gelatin, and sodium alginate to fabricate synthetic blood vessels. Using a stepper motor module and a vertically directed extrusion nozzle, they created hollow tubular scaffolds seeded with mouse epidermal fibroblasts. The composite scaffolds exhibited improved mechanical properties with minimal cytotoxicity from CNT doping.

**Figure 3:**
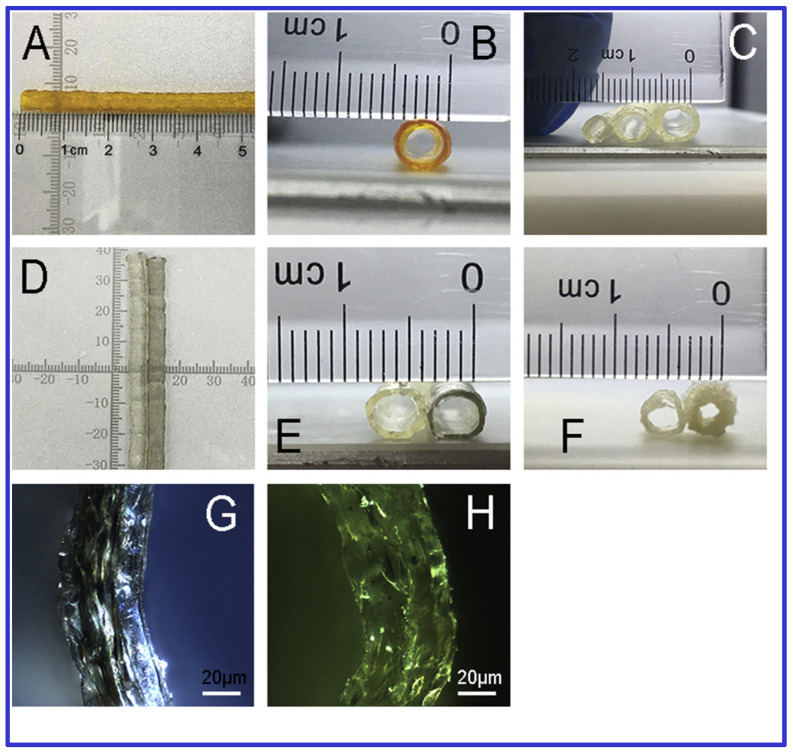
Morphological comparison between polymeric and CNT containing polymeric 3D printed vessel scaffolds. (**A**,**B**) Radial and axial views of printed vessel scaffolds, with an average diameter of 3.5 mm and wall thickness of 0.5 mm. (**C**) Multi-sized tubes with outer diameters of 3, 4, and 5 mm. (**D**,**E**) Macroscopic comparison between scaffolds incorporating carbon nanotubes (CNT) and blank (non-CNT) scaffolds. (**F**) Vessel scaffold structures produced using rotation-axis versus vertical-stacking methods. (**G**,**H**) Polarizing microscope images (10× magnification) comparing Gel-SA-1%C and Gel-SA-0.5%C scaffold groups. Adapted with permission from [25].

Furthermore, 3D Bioprinting has shown promise in treating meniscus and articular cartilage injuries, which current therapies often inadequately address [82]. Szymanski et al. [82] optimized a bioink containing 0.25 mg/mL hyaluronic acid and 0.0625 mg/mL MWCNTs, combined with 2-phospho-L-ascorbic acid, for cartilage repair. The comparison of MWCNT containing hydrogel with blank counterpart is showed in Figure 3. Their results indicated that this combination positively impacted cell viability and gene expression, although a progressive loss in transcriptional activity was noted.

Integrating CNTs into hydrogels for Bioprinting holds substantial promise for advancing TE and regenerative medicine. CNTs enhance hydrogels by improving mechanical strength, electrical conductivity, and cell proliferation. CNTs, classified into SWCNTs and MWCNTs, have properties that influence their classification into metallic, semimetallic, or semiconducting types based on their chiral angle, which ranges from 0 to 30°.

Incorporating CNTs into hydrogels improves flow characteristics and electrical conductivity. For example, Nurly et al. demonstrated that adding CNTs to a double network hydrogel made from polyethylene glycol (PEG) and polyvinyl alcohol (PVA) enhanced printability, viscoelasticity, and shape memory [39]. Deng et al. developed a thermoresponsive, self-healing hydrogel by combining NIPAM, nanoclay (laponite), and CNTs [49]. This hydrogel showed improved mechanical properties, with a Young’s modulus of 46.8 kPa, and exhibited electrical conductivity, making it suitable for sensor applications. Håkansson et al. found that adding less than 6% CNTs by weight to cellulose nanofibril hydrogels significantly increased conductivity from 10^−6^ S/cm to 10^−2^ S/cm [47]. Also, GelMA/DNA-coated MWCNTs demonstrated a 24 ± 1.8 S/cm conductivity and a viscosity suitable for extrusion-based Bioprinting. Even under stress, MWCNTs maintained their electrical conductivity, indicating potential for wearable bioelectronics.

**Figure 4:**
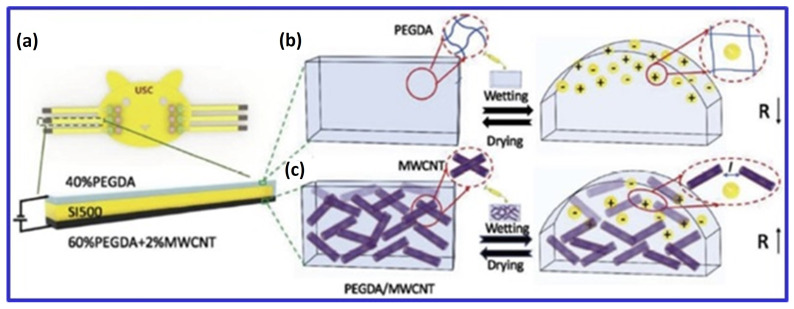
Schematic of a three-layer liquid sensor: (**a**) The sensor includes a non-conductive (SI500) layer, a PEGDA hydrogel layer, and a PEGDA/MWCNT hydrogel layer. (**b**) In wet conditions, the PEGDA layer’s resistivity decreases due to water-facilitated electron transport, while the PEGDA/MWCNT layer’s resistivity increases as MWCNT spacing expands. (**c**) the conductivity of PEGDA/MWCNT composite hydrogel by swelling behavior in wetting and drying state. Adapted from [80].

Three key biomedical applications of 3D bioprinted CNT-containing hydrogels—neural, bone, and cardiac—require specific electrical and mechanical properties. For neural applications, MWCNTs in cellulose-based scaffolds achieved filament widths of less than 1 mm and an electrical conductivity of 0.38 S/cm with 20 wt% CNTs, enhancing cell proliferation and promoting spontaneous neuronal activity [46]. In cardiac applications, Li et al. showed that CNTs improved the mechanical properties of 3D hollow structures and maintained fibroblast, smooth muscle cell, and endothelial cell viability, especially at concentrations below 0.5 wt%; higher concentrations reduced cytocompatibility [25]. Li et al. also reported that the electrical resistance of PEGDA hydrogel decreased with 2 wt% MWCNTs but increased upon swelling due to liquid exposure. Amine-functionalized MWCNTs in PEGDA improved mechanical properties by 189% and enhanced charge storage capacity, promoting cell proliferation and neuronal differentiation under electrical stimulation (Figure 4) [80]. Although research primarily focuses on graphene oxide (GO) and CNTs, studies also explore carbon nanodots and activated carbon in similar applications.

### 3.4. Activated Charcoal

AC is a chemically modified form of amorphous carbon with a microporous 3D structure. It has a high surface area and functionality, allowing it to adsorb various chemical species efficiently. Its large surface area to volume ratio makes it valuable for industrial processes and biological applications, including cholesterol management and toxicant removal [20]. AC also acts as a catalyst in radical-mediated reactions, which are essential for industrial processes such as dye degradation.

It has been established that some of the most important aspects that led to the observed antibacterial activity of AC include its large extent of surface that enables bacterial cells to attach to it quickly and the presence of several groups within AC that are capable of interacting with bacterial membranes [83,84]. These interactions may also damage the bacteria’s cell wall, leading to cell lysis and death. The disk diffusion assay determines the efficacy of antibacterial agents, MIC, and time-kill studies. Some of the chemical changes which intensify antibacterial characteristics include it’s incorporation with silver nanoparticles or other antimicrobial agents. However, limitations may reduce its applicability in specific uses, including the fact that it may develop bacterial resistance and is effective only under certain conditions [83,85].

Hydrogels containing activated charcoal or integrated AC hydrogel systems have shown considerable promise in Bioprinting. These hydrogels significantly improve mechanical properties, biocompatibility, and antibacterial effectiveness. Research has explored how incorporating activated charcoal into hydrogels can enhance printing accuracy, structural strength, and antimicrobial properties. It is crucial to characterize surface characteristics like porosity and functional group density to optimize the antibacterial utility of AC. Using methods such as scanning electron microscopy (SEM) [86], Brunauer-Emmett-Teller (BET) surface area analysis [87], and the Barrett-Joyner-Halenda (BJH) method for pore size distribution analysis, it can be characterized easily.

Additionally, the production processes are important; for example, the physical or chemical activation procedure affects the overall performance of AC. Methods like Fourier-transform infrared spectroscopy (FTIR) for surface functional group analysis and X-ray photoelectron spectroscopy (XPS) for elemental composition determination [86]. In Bioprinting, incorporating additives like AC into hydrogels requires optimizing concentration, crosslinking mechanisms, and rheological properties to balance functional improvements with printability and biocompatibility. Specifically, AC concentration must be carefully controlled to maintain proper rheological properties while achieving desired enhancements [88]. Such bioactive hydrogels are particularly suited for applications like wound healing, where they support cell growth and specialization [89]. Studies have aimed to optimize the concentration of activated charcoal to balance printability with the stability of the printed material.

Activated charcoal’s potential extends to theranostic hydrogels, which combine therapeutic substance delivery with diagnostic imaging capabilities. This highlights the multifunctional potential of AC in developing advanced tissue constructs. Research has predominantly focused on extrusion-based Bioprinting for AC-containing hydrogels. Wu et al. demonstrated the successful creation of 3D hydrogels with various forms using a blend of PNAGA-AMPS/PEDOT/PSS with activated charcoal. Including AC did not negatively affect the biomaterial ink’s printability and endowed the hydrogel with supercapacitor properties, allowing the fabrication of printable electrodes [50]. These hydrogels-maintained cell survival rates between 74% and 100% for L929 fibroblasts, indicating their suitability for various electrical applications, including use as electroactive scaffolds in soft TE.

## 4. Challenges and Future Trends

Carbon-based polymeric nanocomposite hydrogels (NCHs) are at the forefront of biomedical materials research due to their incorporation of nanoparticles such as graphene, carbon nanotubes (CNTs), carbon dots (CDs), and activated charcoal (AC) into polymer matrices. While these advanced materials offer numerous benefits, several significant challenges must be addressed to realize their full potential. Table 3 provides a comprehensive comparison of bioprinting manufacturing techniques, highlighting their cost-effectiveness, speed, resolution, and cellular viability. One of the primary challenges is determining the optimal concentration of nanoparticles within the hydrogel matrix. Although integrating nanoparticles can enhance the hydrogels’ mechanical strength, electrical conductivity, and bioactivity, excessive concentrations can compromise the hydrogels’ printability and structural stability. This can lead to a reduction in their effectiveness for applications such as tissue engineering and drug delivery. Ensuring biocompatibility and safety is another critical concern. High levels of nanoparticles may induce cytotoxicity or provoke inflammatory responses, necessitating rigorous safety evaluations to confirm that these hydrogels are suitable for clinical use. A further technical hurdle is the uniform dispersion of nanoparticles within the hydrogel matrix. Nanoparticles often agglomerate, resulting in inconsistent mechanical properties and diminished functionality. Achieving a homogeneous distribution necessitates advanced mixing techniques and surface modifications to prevent aggregation. Scaling up production while maintaining quality and reproducibility presents another challenge. The successful processes developed at the lab scale may not directly translate to large-scale manufacturing due to differences in processing conditions and material handling. Ensuring these materials can be produced consistently and at scale is crucial for their eventual application in clinical settings.

Lastly, integrating multiple functionalities into a single hydrogel system requires complex material design and synthesis. This demands precise control over the interactions between various components to balance properties like electrical conductivity, fluorescence, and mechanical strength without compromising structural integrity. Future trends in this field will likely focus on overcoming these challenges through innovative research in material design and manufacturing processes. Continued exploration of new nanoparticle types, improved synthesis methods, and advanced fabrication techniques will be essential in addressing these issues and enhancing the capabilities of carbon-based NCHs.

## 5. Conclusions

In conclusion, carbon-based nanocomposite hydrogels signify a major breakthrough in biomedical materials, greatly improving their mechanical, electrical, and biological functionalities. To capitalize on these advancements, focusing on the effective development and application of these hydrogels is imperative. This involves refining the concentration of nanoparticles, verifying their safety for biological use, and ensuring an even distribution throughout the matrix. Successful progress in these areas will enable new and exciting applications in tissue engineering, drug delivery, and regenerative medicine. Continuous exploration and innovation in material composition, along with efficient production methods, are essential for unlocking the full capabilities of these sophisticated hydrogels. By addressing these factors, the field can move toward creating advanced, multifunctional biomedical materials that will enhance healthcare outcomes and contribute to the advancement of biotechnological innovations.

## Figures and Tables

**Table 1:** Key features of carbon-based materials.

Material	Dimensions	Elastic Modulus (TPa)	Electrical Conductivity (Sm^−1^)	Thermal Conductivity (Wm^−1^K^−1^)	Reference
**Carbon nanotubes**					
Single-walled carbon nanotubes (SWCNTs)	Ø = 1–2 nm (Length in µm to mm)	1	~1 × 10^4^	~6 × 10^3^	[2,21]
Multi-walled carbon nanotubes (MWCNTs)	Ø = 5–20 nm (Length in µm to mm)	1.2	~1 × 10^4^	~6 × 10^3^	[22]
**Graphene**	Thickness = 0.34 nm Lateral size = >100 nm Surface area = 2630 m^2^/g	1	6 × 10^5^	5.1 × 10^3^	[23]
**Graphene oxide**	Thickness = 0.4–1.7 nm Lateral size = >100 nm Surface area = 2391 m^2^/g	0.22	1 × 10^−1^	2 × 10^3^	[24]
**Carbon dots**	Size < 20 nm	0.1 to 0.3	~43	~0.605	[25,26,27]

**Table 2:** Summary of Optical Properties and Results for Carbon Quantum Dots. This table highlights various optical properties of carbon quantum dots (CDs) along with their measured results from different studies, demonstrating their effectiveness in applications such as 3D printing, in vivo imaging, and bioimaging diagnostics.

Optical Property	Results	Reference
Fluorescence Emission	Measured fluorescence and quantum yield of carbon quantum dots (CDs) as photocatalysts in 3D printing. They reported high monomer conversion and efficient photoluminescence under visible light, essential for rapid 3D polymerization.	[68]
Fluorescence Emission	In vivo imaging of scaffolds implanted in mouse models, showing the CDs maintained fluorescence and enabled real-time visualization without inducing inflammation.	[63]
Fluorescence Emission, Rheological Characteristics.	CDs in hydrogels demonstrated fluorescence emission when excited at 365 nm. This property is beneficial for diagnostic applications, combining bioimaging with mechanical stability in hydrogels.	[41]
Fluorescence Emission	high photoluminescence with tunable emission properties, which were effectively used in bioimaging applications, enhancing imaging contrast and specificity in medical diagnostics	[55]
Excitation-Dependent Emission	exhibited significant excitation-dependent emission, with fluorescence intensity varying markedly across different excitation wavelengths, demonstrating enhanced brightness and stability under optimal excitation conditions.	[41]

**Table 3:** Comparison of Bioprinting manufacturing techniques categorized by major attributes, including cost-effectiveness, speed, resolution, and cellular viability. Each technique exhibits unique strengths and limitations, with specific requirements for material properties and processing conditions that influence its suitability for various biomedical applications.

Category	Bioprinting Techniques	Key Advantages	Key Limitations	Ref.
Cost-Effective Techniques	Inkjet-based, Extrusion-based	- Low cost - High cellular viability (Inkjet) - Wide range of ink viscosities (Extrusion) - Large construct fabrication (Extrusion)	- Low ink viscosity requirement (Inkjet) - Prone to clogging and cell sedimentation (Inkjet) - Low resolution (Extrusion)	[90,91,92,93,94]
High-Speed Printing	Laser-based, Stereolithography, Digital Light Processing	- High printing speed - Orifice free (no clogging) - High resolution - Complex structure fabrication (Stereolithography, DLP)	- Expensive - Photosensitive polymer requirement (Stereolithography, DLP) - Cytotoxicity risk (DLP)	[92,93,94,95,96,97,98]
Resolution-Focused	Inkjet-based, Laser-based, Stereolithography, Digital Light Processing	- High resolution (1–150 μm) - Precise layer curing (DLP)	- Limited by viscosity and material properties (Inkjet, Laser-based) - Requires optimization (Stereolithography)	[90,91,92,95,96,97,99,100]

## Data Availability

Data are contained within the article.
